# Prostatic Abscess in a Neonate

**DOI:** 10.7759/cureus.12137

**Published:** 2020-12-18

**Authors:** Manikandasamy Veluchamy, Ashiq Zindha Basheer Ahamed

**Affiliations:** 1 Neonatology, Ahalia Hospital, Mussafah, Abu Dhabi, ARE; 2 Radiology, Ahalia Hospital, Mussafah, Abu Dhabi, ARE

**Keywords:** abscess, urinary tract infection, neonate, prostate, pyuria, prostatic abscess, urosepsis, phimosis, hematuria

## Abstract

Urinary tract infection (UTI) is the most commonly occurring serious bacterial infection in young infants. Uncircumcised male infants have a higher rate of UTI when compared with circumcised male infants and girls. A prostatic abscess is a very rare clinical variety of UTI, especially in neonates.

We present the case of a 15-day-old male neonate who developed a rare variety of urosepsis. The baby was evaluated and found to have a prostatic abscess. Ultrasound of the abdomen showed an enlarged prostate gland with diffuse heterogeneous hypoechogenicity. Magnetic resonance imaging (MRI) of the pelvis showed an enlarged, lobulated prostate with T2 hyperintense signal and T1 hypointense signal and diffusion restriction. The post-contrast images in the pelvis-MRI also showed peripheral rim enhancement suggestive of a prostatic abscess. Urine culture showed growth of methicillin-resistant Staphylococcus aureus (MRSA). The baby was treated with intravenous vancomycin, and pus was drained through a transurethral approach.

Phimosis can cause purulence in the prostate. Prostatic abscess usually has a good prognosis in neonates when diagnosed early and appropriate treatment was instituted.

## Introduction

Urinary tract infection (UTI) is the most commonly occurring serious bacterial infection in young infants. UTI in neonates (<30 days of age) is confederated with bacteremia and congenital anomalies of the kidney and urinary tract. The true incidence of UTI in the neonatal age group is not known because the prevalence of bacteriuria in healthy term neonates is very uncertain and difficult to assess. UTI affects approximately one in six febrile neonates ≤30 days of age and the prevalence of UTI in febrile infants <8 weeks of age is 13.6%. UTI in neonates have a male predominance; males are affected 2.5-times greater than females [1,2].

Uncircumcised male infants have a higher rate of UTI when compared with circumcised male infants and girls. The presence of underlying renal abnormalities increases the risk of neonatal UTI. Vesicoureteric reflux is associated with approximately 20% of neonatal cases of UTI. Neonates with UTI often present with fever, poor feeding, lethargy, vomiting, and diarrhea. Neonatal UTIs are sometimes associated with jaundice, also [3].

The occurrence of pyogenic infection of the prostate in a neonate is very rare. We report the case of a prostatic abscess in a 15-day-old neonate, who was treated successfully.

## Case presentation

**Figure 1:**
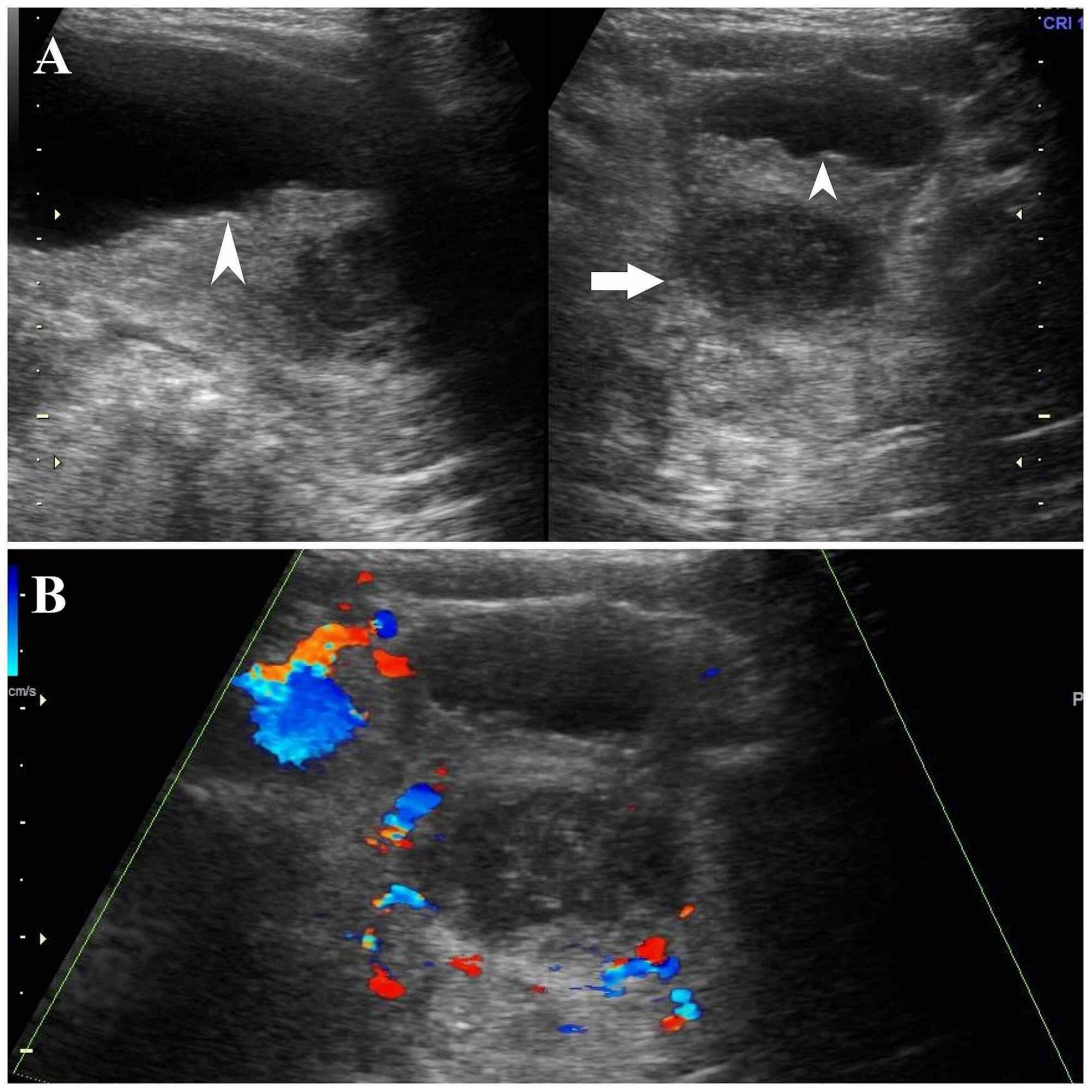
Ultrasound of the urinary bladder and prostate with Doppler imaging A – Arrowheads show prominent wall thickening, and mucosal irregularities are seen along the inferior wall of the urinary bladder. The horizontal arrow shows the enlarged hypoechoic appearance of the prostate gland. B – Colour Doppler imaging of the prostate shows prominent peripheral vascularity with no definite central vascularity.

A 15-day-old neonate was brought to the out-patient department (OPD) with complaints of fever, hematuria, and pyuria. The baby had a temperature of 37.8 degrees Celsius. Urine analysis revealed slightly turbid urine with more than 50 leucocytes per high power field. Abdominal ultrasound was initially performed and revealed an enlarged prostate gland with heterogeneous hypoechoic appearance, prominent peripheral vascularity, and no definite central vascularity on color Doppler imaging (Figure 1). Differentials at this stage included acute prostatitis and prostatic abscess. Associated diffuse thickening and mucosal irregularities along the inferior wall of the urinary bladder were seen suggestive of cystitis. C-reactive protein (CRP) was 50.3 mg/L initially at admission. Rectal examination was done after the baby passed urine, which showed enlarged fluctuant prostate.

**Figure 2:**
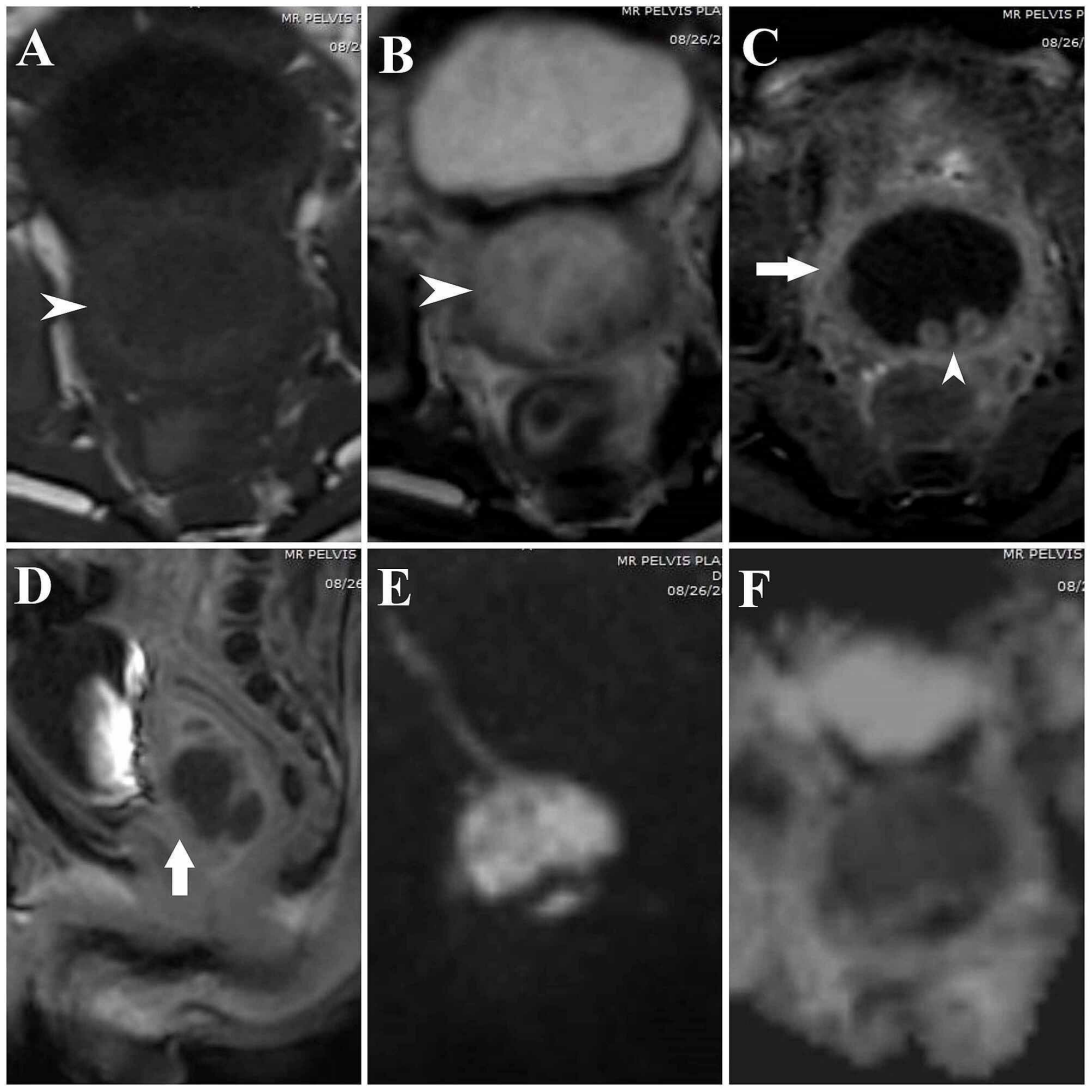
MRI pelvis plain and contrast images A – Axial T1-weighted image, arrowhead shows significantly enlarged and hypointense prostate gland. B – Axial T2-weighted image, arrowhead shows significantly enlarged and hyperintense prostate gland. C – Axial T1-weighted post-contrast image, horizontal arrow shows significant peripheral contrast enhancement of the prostate gland, and the vertical arrowhead shows peripherally enhancing ejaculatory ducts. D – Sagittal T1-weighted post-contrast image shows residual contrast in the urinary bladder. The vertical arrow shows significant peripheral contrast enhancement of the prostate gland. E – Diffusion-weighted image shows hyperintense enlarged prostate gland. F – Apparent diffusion coefficient (ADC) mapping corresponding to image E shows hypointense prostatic parenchyma with a low ADC signal suggestive of restricted diffusion.

Contrast-enhanced MRI of the pelvis was done as further evaluation to differentiate between acute prostatitis and prostatic abscess. MRI revealed the prostate gland to be significantly enlarged in size and showed a large lobulated T2-weighted hyperintense signal and T1-weighted hypointense signal region within measuring about 2.5 (craniocaudal) x 2.3 (transverse) x 1.8 (anteroposterior) cm in size. The volume of this region measured about 5.2 cc. This region showed significantly restricted diffusion with a low apparent diffusion coefficient (ADC) signal. A thin rim of hypointense prostatic parenchyma was noted surrounding this region. On post-contrast images, no enhancement was noted within the above-described region, with significant peripheral contrast enhancement confirming an abscess collection (Figure 2). A few thin enhancing septations were also noted within the lesion. A similar peripherally enhancing region measuring about 6.5 x 6.0 mm in size was also noted in the region of the right seminal vesicle suggesting an extension of the abscess. The prostatic urethra was displaced anteriorly with focal loss of the posterior margin of the prostatic urethra, suggesting likely communication of collection with the urethra. Peripherally enhancing ejaculatory ducts were noted within the posterior third of the abscess. The urinary bladder also showed features of cystitis.

**Figure 3:**
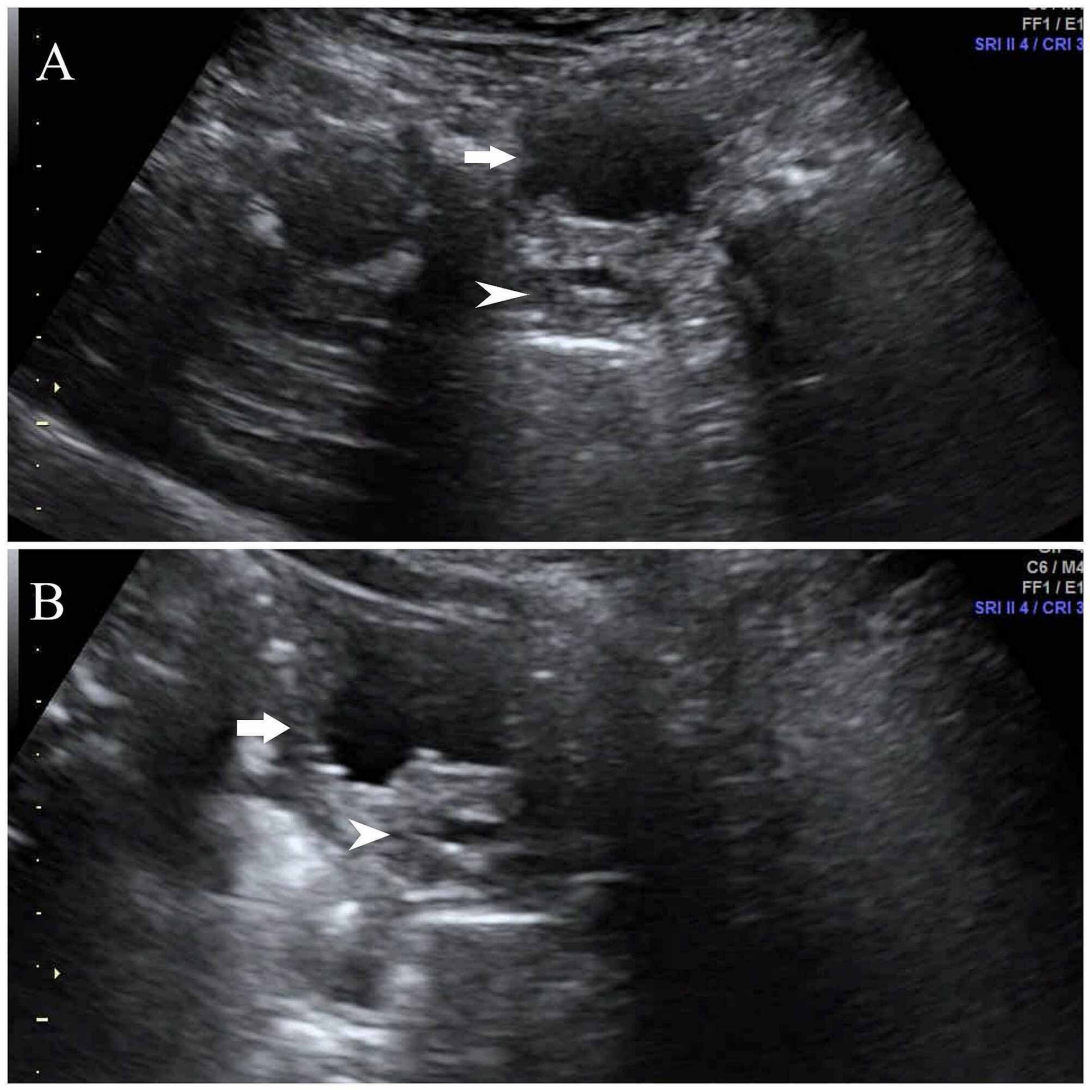
Repeat ultrasound of urinary bladder and prostate after drainage of pus A – Axial image, arrow shows contracted urinary bladder, and the arrowhead shows the tiny central hypoechoic focus of size 6 x 5 mm suggesting residual component. B – Sagittal image, arrow shows contracted urinary bladder, and the arrowhead shows the tiny central hypoechoic focuses suggesting residual component.

The baby was initially started on cefotaxime injections empirically; later, urine culture showed growth of methicillin-resistant Staphylococcus aureus (MRSA), which was sensitive to vancomycin, linezolid, and nitrofurantoin. Then the antibiotic was changed to vancomycin (injection). The prostatic abscess was drained through a transurethral approach. The fever spikes then decreased, and CRP gradually normalized. The ultrasound was repeated, which showed normal-sized prostate with a tiny central hypoechoic focus suggestive of the residual collection (Figure 3). Initially, the baby received cefotaxime injection for four days, and after the urine culture report, vancomycin injections were given for 10 days, and then he was discharged from the hospital. The repeat urine culture after the antibiotic course was sterile. The baby was examined on follow-up and was found to be thriving well.

## Discussion

Urinary tract infections in infants are common, but prostatic abscess is not a common clinical entity in infants. A prostatic abscess is a very rare clinical variety of UTI, especially in neonates. Only 13 cases have been reported in the literature before our case report. Some factors are responsible for the occurrence of prostatic abscess in neonates. The first factor is that neonates, themselves, are at risk of any infection. The second factor is an obstruction to the urinary flow because of physiologic phimosis. The third factor is the presence of squamous metaplasia of the epithelium of the utricle, prostatic urethra, and prostatic glands at birth, which undergoes regression during the first week of life [4].

Two mechanisms can explain the occurrence of prostatic abscess. The first mechanism is the hematogenous spread of organisms to the prostate, most commonly after Staphylococcal bacteremia [5]. The other mechanism is ascending UTI, which may occur because of the reflux of urine from the urethra into the prostatic ducts during micturition, and the most common pathogens causing prostatic abscess by this mechanism are Escherichia coli and other Enterobacteriaceae, like Serratia [6].

Neonates with prostatic abscess usually present with fever, hematuria, or pyuria. The clinical diagnosis of prostatic abscess in neonates is difficult because rectal examinations are not routinely performed in neonates; also, only one-third of neonates present with flocculence. Even though ultrasound imaging helps diagnose a prostatic abscess, MRI is the investigation of choice, especially MRI with contrast, which will differentiate between acute prostatitis and prostatic abscess. Staphylococcus aureus is the most commonly encountered pathogen in a neonatal prostatic abscess.

Treatment of prostatic abscess mainly focuses on the drainage of pus by transurethral, transperineal, or transrectal approach plus appropriate antibiotic therapy based on the culture and sensitivity reports [7].

Williams and Martins reported a series of cases of neonates with prostatic abscesses or periprostatic hematoma. Three cases were of prostatic abscesses, and two had a periprostatic hematoma. Retention of urine was the predominant clinical feature in all those neonates. The diagnosis was easily made by rectal palpation after the bladder had been emptied by a catheter. Escherichia coli and Staphylococcus species were the predominant organisms grown in culture. Urinary retention was relieved by drainage of abscess or hematoma cavity through the transperineal route; the prognosis is good if the abscess cavity is adequately drained [8].

Stewart Mann reported three cases of acute staphylococcal prostatic abscess presented with urinary obstruction in neonates. Urethrogram done in these cases demonstrated enlarged, distended prostate causing obstructive uropathy [4].

Heyman and Lombardo reported a case of metastatic prostatic abscess in a 34-day-old infant who developed staphylococcal pneumonitis and enteritis during the first admission and later developed funiculitis with epididymo-orchitis and urinary retention. Rectal examination revealed enlarged fluctuant prostate. The authors attempted a cystoscopic examination but were unable to pass the cystoscope beyond the prostatic urethra. The cystoscope punctured the prostate, which was followed by a large volume of purulent drainage from the urethra. Culture of the purulent drainage showed growth of Staphylococcus aureus. The infant improved with antibiotic therapy [9].

Collins also reported a case of prostatic abscess in a 10-day-old neonate who died within 36 hours of presentation because of this fulminant urosepsis. A prostatic abscess was found in the autopsy, and culture showed growth of Serratia marcescens [7].

## Conclusions

A prostatic abscess is a very rare entity of urosepsis in neonates. A prostatic abscess usually presents with urinary retention, fever, or pyuria. Imaging modalities like ultrasound or magnetic resonance imaging help in the early diagnosis of a prostatic abscess. Treatment of prostatic abscess involves drainage of pus with appropriate antibiotic therapy. A prostatic abscess usually has a good prognosis in neonates when diagnosed early and if appropriate treatment was instituted.
